# Early Dalmatian farmers specialized in sheep husbandry

**DOI:** 10.1038/s41598-023-37516-z

**Published:** 2023-06-26

**Authors:** A. Sierra, M. Balasse, S. Radović, D. Orton, D. Fiorillo, S. Presslee

**Affiliations:** 1 AASPE «Archéozoologie, Archéobotanique: Sociétés, Pratiques, Environnements» CNRS, MNHN, 75005 Paris, France; 2 Departament de Prehistoria, Facultat de Lletres, Universitat Autonoma de Barcelona, Bellaterra, Barcelona Spain; 3 Croatian Academy of Sciences and Arts, Institute for Quaternary Palaeontology and Geology, Ante Kovačića 5, 10000 Zagreb, Croatia; 4 BioArCh, Department of Archaeology, Environment Building, University of York, Heslington, York, YO10 5NG UK; 5 Department of Chemistry, University of York, Heslington, York, YO10 5NG UK

**Keywords:** Anthropology, Archaeology, Cultural evolution

## Abstract

The spread of farming in the central and western Mediterranean took place rapidly, linked to the Impressa Ware. The Impressa Ware originated somewhere in the southern Adriatic and spread westwards across the Mediterranean. These early farmers had an economy based on cereal agriculture and caprine husbandry, but there is still little information on how this agropastoral system functioned. This study aims to unravel the farming practices of the early Dalmatian farmers linked to the Impressa culture by using an integrated analysis, combining archaeozoology, palaeoproteomics and stable isotopes, applied to the faunal assemblages of Tinj-Podlivade and Crno Vrilo. The results show: (1) the composition of the flocks was overwhelmingly sheep; (2) sheep exploitation at both sites was similar, focusing on milk and meat; (3) sheep reproduction was concentrated at the beginning of winter, with no reproduction in autumn as in later sites in the western Mediterranean. We conclude that a common animal economy existed at both sites, which could be related to the mobility practiced by these early farming societies throughout the Mediterranean.

## Introduction

The spread of the Neolithic in Europe has given rise to very different agropastoral systems throughout time and space in response to environmental and sociocultural factors. The primary spread of these practices across Europe involved two main routes: one around the central and western Mediterranean coast, linked to Impressa pottery, and another through the Danube basin into central Europe, linked to Starčevo–Körös–Criş (SKC) and eventually Linearbandkeramik (LBK) pottery^1^. The major differences between these two primary routes have been highlighted^2,3^ but variability within each of those has not been fully explored. This necessitates, beyond the constitution of the herds, describing carefully how these animals were managed, including the orientation of production as revealed by mortality profiles and the seasonal rhythms of these pastoral systems.

The Adriatic is a key area for the neolithization of the central and western Mediterranean. The 'maritime' stream, linked to the so-called Impressa Ware, first became clearly distinct in this area. This style originated in the southern Adriatic somewhere between Tavoliere and Dalmatia^4^ and its dissemination was fast, with contemporary dates from the beginning of the 6th millennium cal BC on both coasts^5,6^. Different dynamics have been observed for the eastern Adriatic, depending on the previous presence of hunter-gatherers^7^. While in the north (Istria) and south (southern Dalmatia) the occupation of caves previously occupied by hunter-gatherers has been detected, in central Dalmatia the presence of abundant open-air settlements has been documented. Despite this, most of the neolithisation models put forward^5,7,8^ agree that the colonizing groups played an important role in establishing the Neolithic way of life.

The Impressa Ware cultural complex is characterized by a mixed economy based on cereal agriculture^9^ and livestock farming dominated by caprines and with little importance of hunting^2,3,10–13^. Within the caprine category, most archaeozoological work has emphasised a predominance of sheep over goats in Impressa herds^14,15^. However, because these two species have different adaptations and potential, it is necessary to define more precisely the ratio of sheep to goats in these herds. New methods, such as improved osteological criteria^16,17^ and palaeoproteomic analyses^18–20^, provide the opportunity to define with greater certainty the extent to which sheep predominate. The application of Zooarchaeology by Mass Spectrometry (ZooMS) allows sheep and goat to be differentiated due to a difference in peptide markers between the two species^19^. In addition, a better knowledge of the composition of the herds also allows a better characterisation of how the animals were reared. One of the important aspects is demographic management, including reproductive management, which is entirely related to the production strategy and the seasonal management of production. The application of stable isotope analysis to investigate sheep birthing season is a way to approach the rhythms of these systems. Although autumn lambing has been demonstrated in the northwestern Mediterranean at sites post-dating the Impressa^21–23^ (Cardial complex), in stark contrast to spring lambing elsewhere in Europe^24^, this key parameter had not yet been explored in the Impressa.

## Materials

### Sites

**Figure 1:**
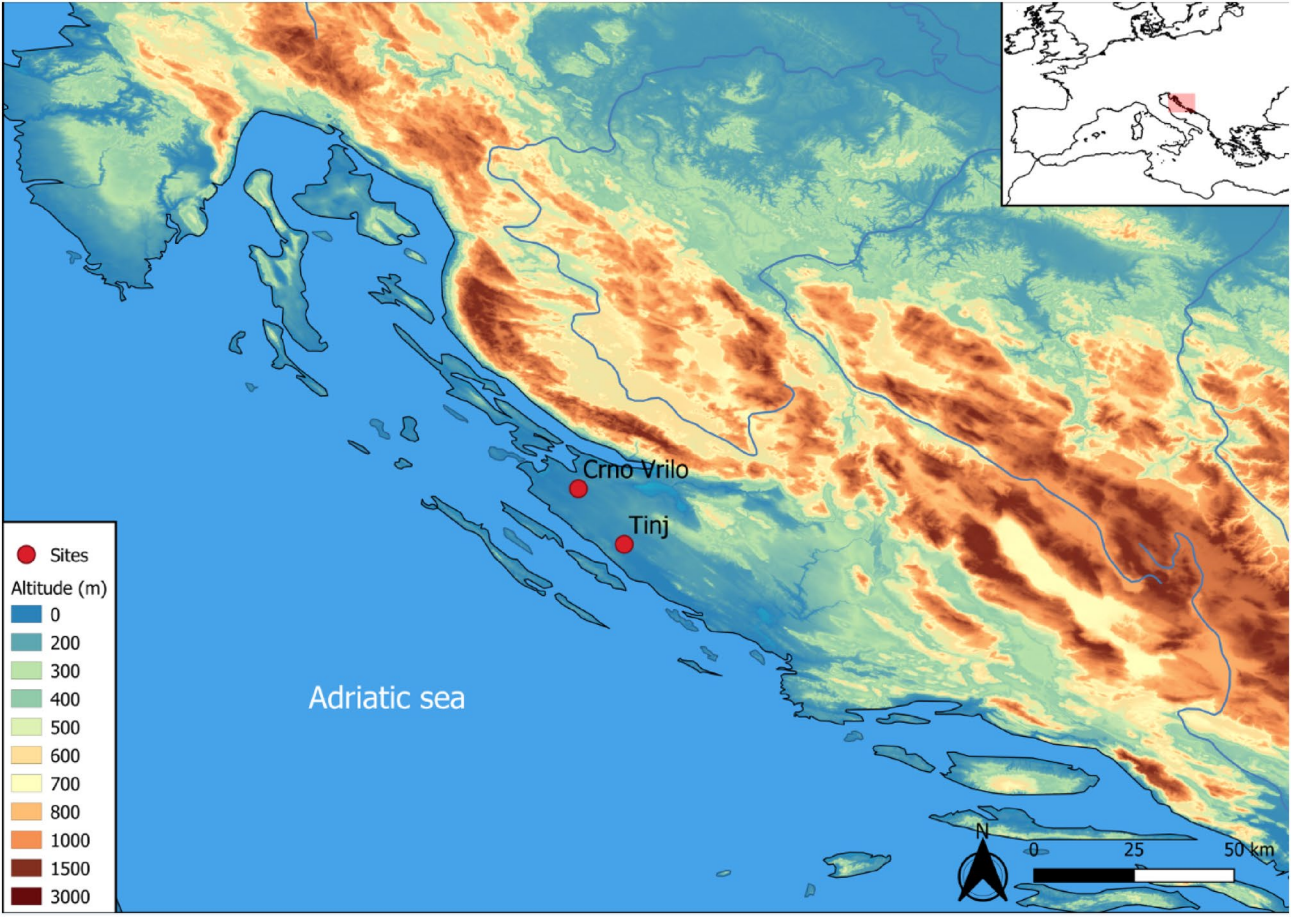
Map showing the location of Tinj and Crno Vrilo in the Eastern Adriatic Sea. Map produced using QGis 3.10.4

The eastern Adriatic coast is a region bounded to the west and south by the Adriatic Sea, and to the north and east by the Dinaric Alps which separate it from the rest of the Balkan Peninsula It is a karst landscape dominated by mountains rising steeply from the coast. In the middle is the Ravni Kotari region, which consists of an alternating series of valleys and low ridges, where farmers have settled since the early Neolithic^25^. In this area are the Tinj-Podlivade and Crno Vrilo sites, among the most important open-air sites in the Adriatic, with the presence of Early Neolithic occupations, and whose faunal collections are among the most abundant and best-preserved (Fig. 1).

Tinj-Podlivade is located in a secondary basin between the Vrana depression to the southwest and mountain range to the northeast, at an altitude of 50 m above sea level. The excavation was carried out by a British-Yugoslav team in 1984^26^. The site occupied an area of 2.8 ha. Two sectors (A and B) were excavated and a stratigraphic sequence of 0.75 m in thickness was documented, with built structures and four pits. Three conventional radiocarbon dates have dated the occupation of this site to the beginning of the 6th millennium BC (5815–5185 cal BC), but with a high standard deviation^18^. The present study has re-dated the sheep T Ovis 44 which confirms the antiquity of the site (ICA-14C-6145: 6900 + /− 60 BP; 5908–5666 calBC at 95.4% probability). The major component of the ceramic assemblages is pottery with impressed decoration. Agriculture is also well documented from the cultivation of three different species of cereals^27^ (barley, emmer and einkorn). In addition, a significant number of weed seeds were documented. The 15,365 recovered faunal remains stand out as undoubtedly one of the most quantitatively important in this geographical area for this chronology. Of these Schwartz^28^ identified a total of 3212 remains. The assemblage is mainly composed of caprine remains (91.4%), with the sheep/goat ratio not being given. Among the other domestic species recovered, remains of cattle, pigs and dogs are also recorded, but in small numbers. Hunting of wild resources is a minor component (included birds and molluscs). These data have led to propose that the economic model of the site was a lowland mixed farming economy ^26^.

Crno Vrilo is located on the right side of the Miljašić Jaruga River, about 12 km from the modern town of Zadar. It was excavated between 2001 and 2005 by members of the University of Zadar^25^. The Neolithic settlement was located 63 m above sea level and covered an area of 6750–7500 m^2^, of which 550 m^2^ were excavated. The cultural layer starts at the surface and has an average thickness of about 0.6 m, overlying the bedrock. The deposits were divided into several excavated layers, but it is regarded as a single-layered site with only a single cultural phase^25^. Excavation has documented constructional remains of dwellings (hearths, ovens, stone walls and posts). Available dating also places it in the Early Neolithic period, with dates around the first half of the 6th millennium BC^25^. The material culture is very rich, with abundant remains of pottery with impressed decoration, flint and bone tools. Archaeobotanical data show a predominance of cereals, followed by leguminous plants (*Fabaceae*). Emmer and einkorn wheat (*Triticum dicoccon* and *Triticum monococcum*) and barley (*Hordeum vulgare*) are documented^29^. From an archaeozoological point of view, the site is very rich, dominated by mammals (NISP = 3564) and molluscs (n = 4217). Among the marine fauna, molluscs stand out, with a wide variety of species such as the mussel (*Mytilus galloprovincialis*) and the oyster (*Oestra edulis*)^30^. Among the macromammals, the largest component is also sheep and goat (95.94%)^31^, with the presence of cows, pigs and wild mammals being very low. The abundance and variety of bird species is also noteworthy. In short, Crno Vrilo would have a mixed farming economy in which the surrounding alluvial areas would be exploited ^25^.

### Samples

#### Mortality profiles and ZooMS analysis

For the elaboration of the Tinj and Crno Vrilo mortality profiles, the mandibles and the loose lower dental remains were used. In the case of Tinj, all the dental remains were analysed. For Crno Vrilo, only the material from Trench A was analysed due to the large size of the total faunal assemblage. All caprine teeth and mandibles from this trench have been studied in detail by one of the authors (S.R.) during his PhD thesis^31^. For the purpose of this study, we have rechecked all original identifications in terms of taxa and age assessment. Only some minor inaccuracies were noted and corrected.

The dental remains of caprines were analysed trying to separate sheep and goats morphologically whenever possible^16,17,32–35^. Among a total of 428 mandibles and dental remains, 193 were selected for ZooMS analysis, including 122 from Crno Vrilo and 71 from Tinj. Of these, 164 correspond to all remains certainly belonging to distinct individuals and morphologically identified as sheep, goat or caprine. A further 29 loose teeth were also selected to corroborate that there was no different representation between these elements.

#### Stable oxygen isotope analysis

A total of 23 lower third molars were selected for isotope analysis, out of the remains attributed to sheep according to the morphological criteria mentioned above, and confirmed as *Ovis aries* by ZooMS analysis. Sixteen samples are from Crno Vrilo and 7 are from Tinj. Most of them were slaughtered between 24 and 72 months (Table 1).

**Table 1:** Samples selected for isotopes analysis.

ID	Species ZooMS	Tooth	Side	Age class (*) (*according to Payne 1973)	Estimated age (*) (*according to Payne 1973)	^14^C date
T63 Ovis	*Ovis aries*	M_3_	R	E	2–3 y	
T9 Ovis	*Ovis aries*	M_3_	R	E	2–3 y	
T76 Ovis	*Ovis aries*	M_3_	L	G	4–6 y	
T8 Ovis	*Ovis aries*	M_3_	L	G	4–6 y	
T48 Ovis	*Ovis aries*	M_3_	L	G	4–6 y	
T47 Ovis	*Ovis aries*	M_3_	L	G	4–6 y	
T44 Ovis	*Ovis aries*	M_3_	R	G	4–6 y	5908–5666 calBC
CV32 Ovis	*Ovis aries*	M_3_	L	D	12–24 m	
CV136 Ovis	*Ovis aries*	M_3_	L	E	2–3 y	
CV129 Ovis	*Ovis aries*	M_3_	L	E	4–6 y	
CV125 Ovis	*Ovis aries*	M_3_	L	F	3–4 y	
CV137 Ovis	*Ovis aries*	M_3_	L	F	3–4 y	
CV113 Ovis	*Ovis aries*	M_3_	L	F	3–4 y	
CV149 Ovis	*Ovis aries*	M_3_	L	F	3–4 y	
CV69 Ovis	*Ovis aries*	M_3_	L	G	4–6 y	
CV52 Ovis	*Ovis aries*	M_3_	L	G	4–6 y	
CV72 Ovis	*Ovis aries*	M_3_	L	G	4–6 y	
CV122 Ovis	*Ovis aries*	M_3_	L	G	4–6 y	
CV97 Ovis	*Ovis aries*	M_3_	L	G	4–6 y	
CV41 Ovis	*Ovis aries*	M_3_	L	G	4–6 y	
CV112 Ovis	*Ovis aries*	M_3_	L	G	4–6 y	
CV43 Ovis	*Ovis aries*	M_3_	L	G	4–6 y	
CV42 Ovis	*Ovis aries*	M_3_	L	G	4–6 y	

## Results

### ZooMS results

**Figure 2:**
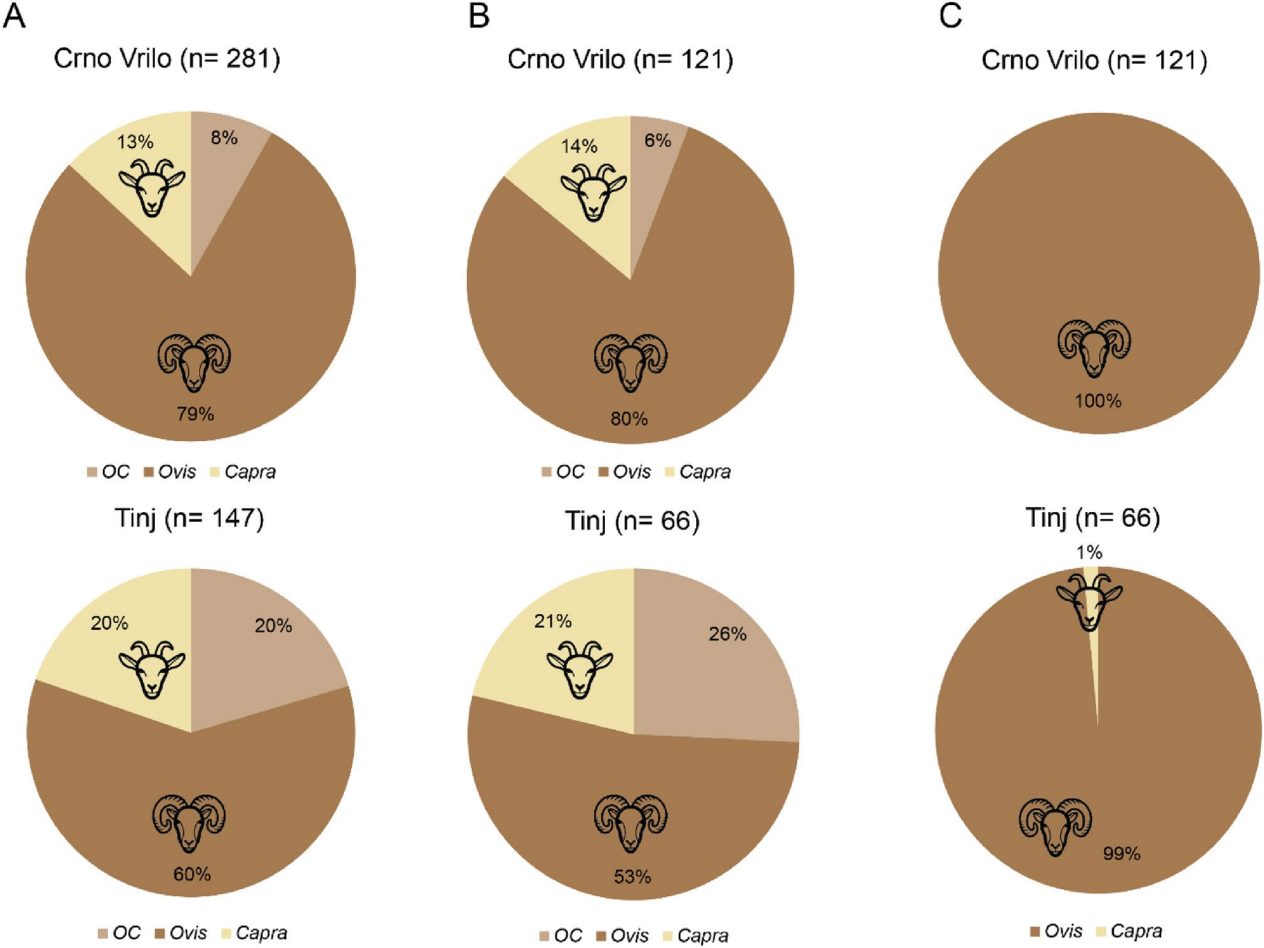
Comparison of the results of sheep and goat identification by site. (**A**) Morphological identification of all analysed remains. (**B**) morphological identification of the remains analysed by ZooMS. (**C**) identification by ZooMS.

All dental remains were analysed using morphological criteria prior to applying the ZooMS methodology, trying to separate sheep and goats^16,17,32–35^. A total of 428 mandible and teeth (including 281 from Crno Vrilo and 147 from Tinj) were analysed (Fig. 2). From osteological criteria, a majority of these remains were attributed to sheep (79% at Crno Vrilo and 60% at Tinj) and a minority to goats (13% at Crno Vrilo and 20% at Tinj). However, a significant percentage of remains could not be classified (8% at Crno Vrilo and 20% at Tinj). This was because they were loose teeth on which the reliability of the identification criteria is lower^16^, especially the fourth deciduous premolar (dP_4_).

Among the 193 samples analysed with ZooMS, 187 were positive and could be separated into sheep and goats. Only 6 were negative due to poor preservation. Out of 187 positive samples (132 being previously identified as sheep, 31 as goats and 24 were unidentified), 186 were identified as sheep and only one—from Tinj—as a goat.

If we compare the results of the morphological identification with those of ZooMS, we can see that although the archaeozoological methods allow us to identify the predominance of sheep, goats tend to be over-represented. In addition, the limitations of the methods (fragmentation, loose teeth, teeth with poorly discriminating criteria, etc.) leave a percentage of individuals unclassified that ZooMS allows us to identify.

No differences were observed between loose teeth and mandibles in those teeth that were misidentified (Table 2). In terms of individual teeth, the P4 generated problems in identifying sheep and goats, with 47.1% of the remains being misidentified. The dP_4_ also generated identification problems, 37.7% of the remains could not be identified, most of them loose. As for molars, only 21.4% have been misidentified.

**Table 2:** Morphological identification by type of remains of samples identified as *Ovis* by ZooMS.

	Loose	Mandible	dP_4_	P_4_	M_1-3_
*Ovis*	67	65	38	18	103
*Capra*	13	18	0	16	24
*Ovis/Capra*	19	5	23	0	4
%misidentified	32.3%	25.3%	37.7%	47.1%	21.4%

Left: comparison of mandibles and loose teeth. Right: comparison by teeth (count has been made for each tooth of the jaw, so their sum is greater than the total number).

### Mortality profiles

**Figure 3:**
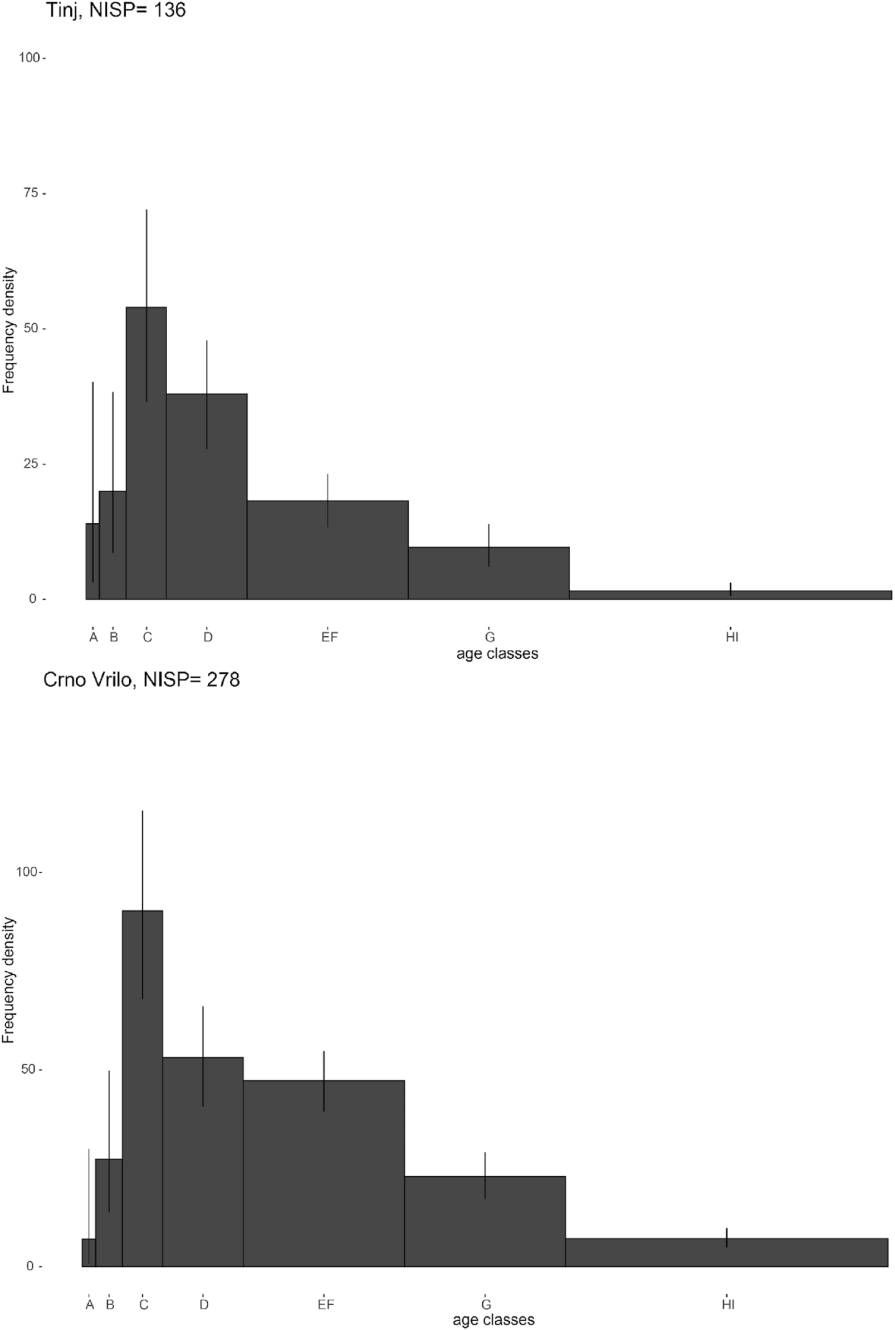
Mortality profiles in Tinj and Crno Vrilo, based on NISP, with 95% credible intervals of the frequency density (see ^30^). Age classes: A (0–2 months), B (2–6 months), C (6–12 months), D (12–24 months), EF (24–48 months), G (48–72 months) and HI (> 72 months).

Since 99% of the remains analysed with ZooMS belong to sheep, it has been assumed that most of the teeth belong to sheep and the mortality profile has been performed using all the teeth (Fig. 3). The mortality profile of Tinj is characterised by the presence of juveniles and young adults, between 6 and 24 months (age classes C to D). On the other hand, there is also the presence of adult classes, between 24 months and 6 years (age classes EF to G). Finally, the low presence of the youngest classes, which correspond to animals aged between 0 and 6 months, is noteworthy. The absence of these animals may be due to problems of preservation of these remains as they are younger or because the animals could birth in another area.

The caprine mortality profile of Crno Vrilo is very similar to that of Tinj. Dead animals between 6 and 12 months are very abundant. Other very frequent age classes are D, between 12 and 24 months, and EF, between 24 months and 4 years. Finally, like Tinj, there is a noteworthy absence of the younger classes, due to possible reasons mentioned above. In any case, the presence among the bones of some perinatal animals confirms their presence at the site^31^.

### Stable isotope analysis

#### Oxygen isotope ratios

**Figure 4:**
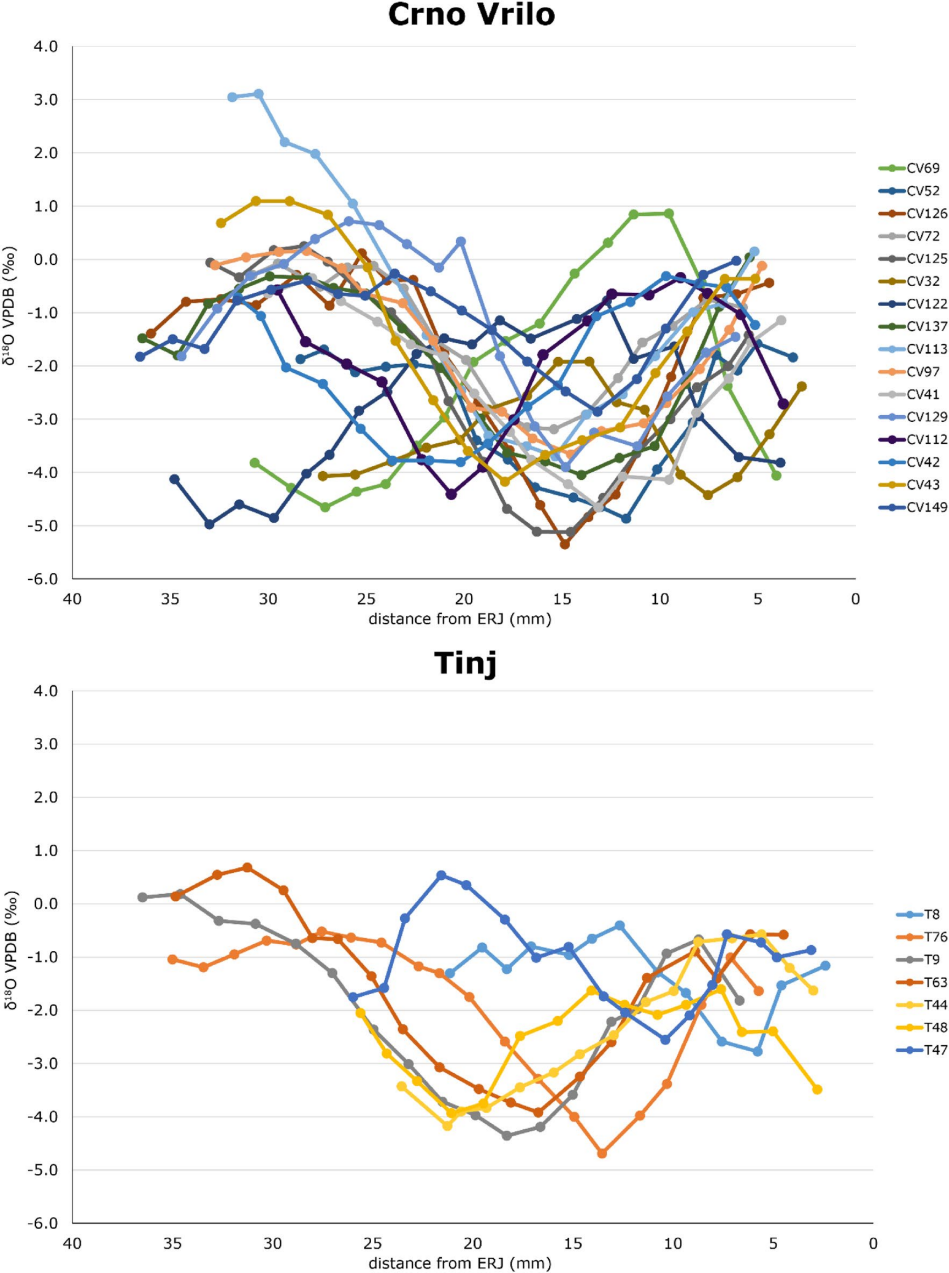
Results from the sequential stable oxygen isotope analysis in third molar (M3) enamel bioapatite. Each sample is located in the tooth crown relative to its distance from the enamel-root junction (ERJ).

The results from the measurements of stable oxygen isotope ratios are shown in Table 3 and Fig. 4. Overall, the δ^18^O values vary between − 5.4 and 3.1‰. The mid-range δ^18^O value (max + min/2) varies between − 3.6 and 0.3‰, and the amplitude of intra-tooth variation is between 3.4 and 5.7‰. The intra-tooth δ^18^O sequences measured in the M3 vary according to a sinusoidal pattern, which probably reflects the seasonal cycle, with the lowest values in the cold season (winter) and the highest in the warm season (summer).

**Table 3:** Results from stable oxygen (δ18O) analysis of enamel bioapatite.

		d^18^O_VPDB_			
	n	Max	Min	Mid-range	Amplitude
T Ovis 76	20	− 0.52	− 4.7	− 2.60	4.17
T Ovis 8	13	− 0.41	− 2.78	− 1.59	2.37
T Ovis 48	15	− 1.60	− 3.93	− 2.77	2.33
T Ovis 47	17	0.54	− 2.55	− 1.01	3.09
T Ovis 44	15	− 0.57	− 4.17	− 2.37	3.60
T Ovis 63	19	0.68	− 3.92	− 1.62	4.60
T Ovis 9	18	0.18	− 4.36	− 2.09	4.54
CV Ovis 69	17	0.86	− 4.65	− 1.90	5.52
CV Ovis 52	18	− 1.58	− 4.87	− 3.22	3.29
CV Ovis 136	21	0.11	− 5.35	− 2.62	5.46
CV Ovis 72	17	− 0.08	− 3.19	− 1.63	3.11
CV Ovis 125	19	0.25	− 5.12	− 2.44	5.37
CV Ovis 32	16	− 1.92	− 4.43	− 3.17	2.51
CV Ovis 122	20	− 0.78	− 4.97	− 2.88	4.20
CV Ovis 137	19	0.04	− 4.05	− 2.00	4.09
CV Ovis 113	17	3.11	− 3.70	− 0.30	6.81
CV Ovis 97	18	0.16	− 3.66	− 1.75	3.82
CV Ovis 41	17	− 0.35	− 4.66	− 2.51	4.31
CV Ovis 129	18	0.72	− 3.90	− 1.59	4.62
CV Ovis 112	16	− 0.34	− 4.41	− 2.38	4.07
CV Ovis 43	16	1.10	− 4.17	− 1.54	5.27
CV Ovis 42	17	− 0.31	− 3.81	− 2.06	3.50
CV Ovis 149	19	− 0.03	− 2.86	− 1.45	2.83
Min		− 1.92	− 5.35	− 3.64	3.43
Max		3.11	− 2.55	0.28	5.66

Specimen, tooth, maximum (max) and minimum value (min), mid-range and amplitude of intra-tooth variation (A).

#### Modelling of the δ18O sequences

**Figure 5:**
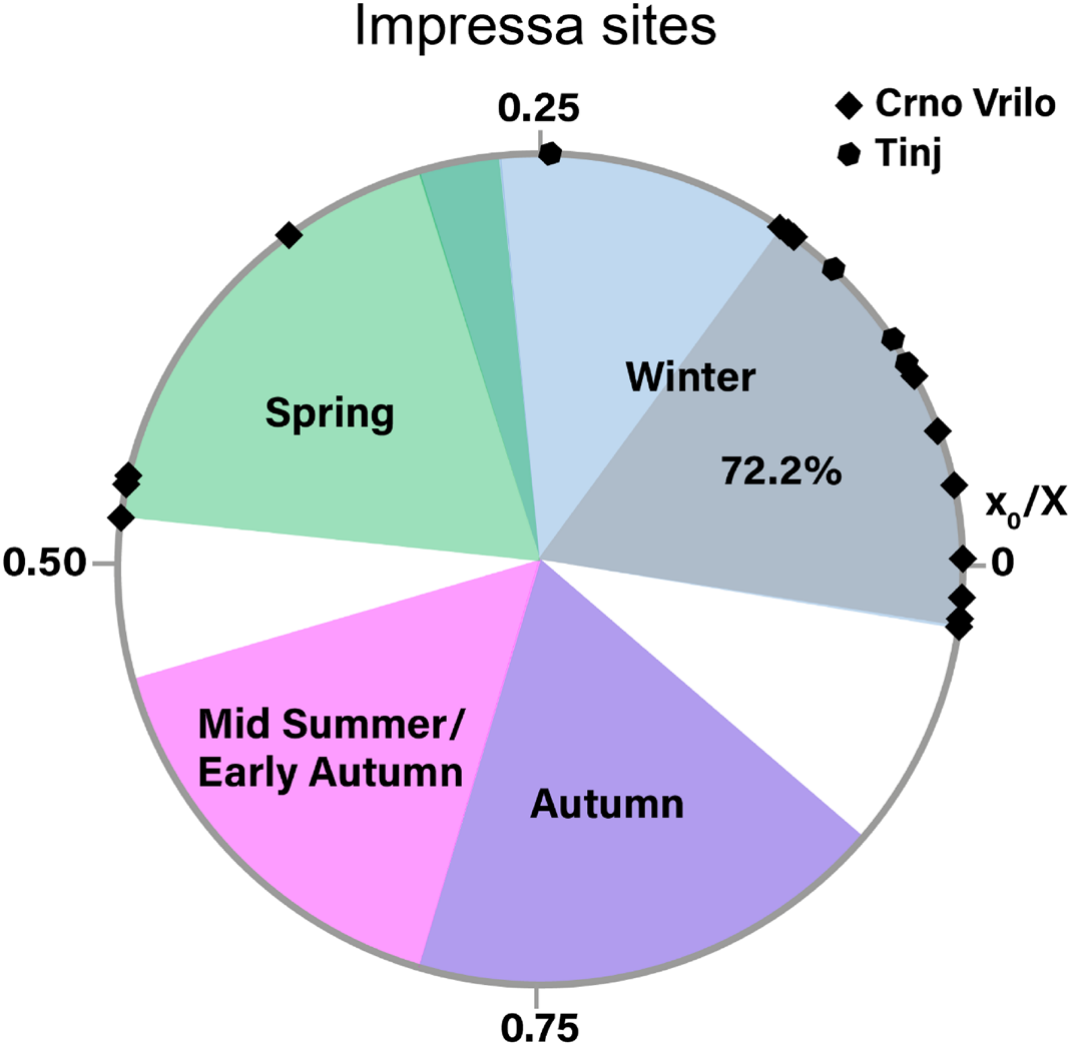
Distribution of sheep births at Tinj and Crno Vrilo, as reflected by the position of the maximum δ^18^O value in tooth crown (× 0) normalized to the period of the cycle (X). The birth season is compared with modern reference sheep (Carmejane CAR^36^; Rousay ROU^24^, Le Merle and La Fage^37^). Blue, green, purple and pink color areas represent × 0/X ratios obtained from modern specimens.

Results from the modelling of the δ18O sequences and the normalized location in tooth crown of the δ18O sequence optimum (x_0_/X) are shown in Fig. 5. T8 Ovis, T48 Ovis, T47 Ovis, CV32 Ovis and CV149 Ovis could not be modelled because their sequences were truncated. At Tinj, x_0_/X ratios for 4 sheep vary between 0.08 and 0.25, defining a breeding period of 0.17 year (around 2 months). At Crno Vrilo, the length of the breeding period, estimated from the analysis of 14 sheep, is 0.5 years (around 6 months), with x_0_/X ratios varying between 0.98 (or − 0.02) and 0.48. However, at this site, most individuals (n = 10) have x_0_/X ratios between 0.98 and 0.15, defining a main breeding period of 0.17 years (2 months).

## Discussion

### Sheep specialization

The application of paleoproteomic identification (ZooMS) to separate sheep and goats—the first such application for the Adriatic region—has shown that practically all the specimens analysed are *Ovis aries*. This indicates that the early Dalmatian herds were composed almost entirely of sheep, implying a strong specialization in sheep husbandry among the early Adriatic farming communities. If this is the case in Dalmatia at such an early date and close to the beginning of the Impressa current, one wonders to what extent this could be the norm during the Neolithic in other nearby regions. Moreover, taking into account the results of this work showing the over-representation of goats and the loss of information due to the lack of classification of some individuals, one might also wonder whether the sheep/goat ratios for the period are at all reliable. The results have shown that teeth such as dP_4_ and P_4_ generate problems in separating sheep and goats. These problems have already been pointed out by previous studies, especially for dP_4_^16^. Therefore, these problems should be taken into account when separating sheep and goats, in order to avoid introducing a bias in the interpretation of flock composition in the past.

Specialization in particular livestock has both economic and social implications. Halstead^38^ has proposed that livestock farming focused on a single species reduces the security of the herd and the variety of available products. In addition, specialization would be linked to large-scale herding^38^ and even altitudinal mobility for the Mediterranean^39^. But, why would the first farmers have such heavily sheep-based husbandry?

One of the first reasons we could propose is ecological. The Mediterranean environment has particular characteristics, with mild rainy winters and hot and dry summers^40^. This could have made difficult the adaptation of some domestic species with higher resource requirements for their survival, such as cattle. However, some of the recent meta-analyses conducted on the Balkan and Mediterranean region have shown that variation in animal management could not be explained by adaptation to the environment alone^3^, and environment cannot explain the preference for sheep over goats.

Another explanation for this specialization, related to the previous one, could be economic. The sheep could have been a very important animal economically for the first farmers. This species provides a multitude of products with higher nutritional values than goats^41–43^. This would lead to a preference in the first herds over the latter species.

In addition, sheep-centered animal husbandry may have social implications. Animal resources can become vehicles of enduring social relations^44^, and may have a social value that goes beyond the economic one^45,46^. This social value of sheep may have been present in Adriatic farming societies, explaining the specialisation in this species. This special value is difficult to unravel with current data, but the appearance in the subsequent Danilo period of objects such as rhytons (zoomorphic-shaped vessels documented from the Middle Neolithic onwards) may be indicators of the importance of sheep beyond the economic role^47^.

Finally, specialisation could be explained by the way the farming economy spread in the northern Mediterranean. Specialization in sheep has been observed also in sites related to Impressa pottery in Italy and southern France^14,48^, so it seems to be linked with the early Neolithic communities of the central and western Mediterranean. Current archaeological data show a rapid diffusion of farming communities related to the Impressa complex^45^. This rapid diffusion must have taken place by sea^46,49^ using boats of different types. This navigation would be supported for the Adriatic case by the occupation of the Adriatic islands^50^ and by the presence of artifacts made with flint from Gargano, southern Italy^51,52^. Navigation has also been previously documented for the settlement of Cyprus^53^ and the island of Crete^54^. Therefore, sheep specialization may have had to do with an anticipatory mobility strategy^55^ in which human communities carried out a strategy adapted to navigation^10,56,57^. Both sheep and goats could be the perfect animal to transport in a first colonization because of their light weight, reproductive capacity and the variety of products they can bring to these communities, but, in addition, the docility of sheep could have played a role in their favour. Thus, building on the argument of Zilhao^49^ for caprines in general, the first farmers could have focused on one species with many advantages for both travel and settlement to increase the chances of successful travel.

Any of these proposals could be plausible with the present data, and several or all of them could have occurred together. In this respect, the continuity of specialization in caprines (possibly sheep) in Dalmatia beyond the early Neolithic is striking^2,3^.^.^ The initial specialization could be explained by the Neolithic diffusion, as observed in Greece in previous centuries, where livestock farming was very much centred on caprine animals (especially sheep), but cattle and, above all, pigs were more important in quantitative terms than in Dalmatia^58^. This importance would acquire social and/or ideological aspects in later periods, as demonstrated by the widespread diffusion of zoomorphic (*rhyton*) vessels from the Middle Neolithic onwards^47^.

### Sheep products

The combination of ZooMS and mortality profiles has allowed us to obtain reliable mortality profiles for sheep. The mortality profiles of Tinj and Crno Vrilo show the predominant culling of young animals, between 6 and 24 months of age, but also the maintenance of quite a few animals beyond 24 and even 48 months. These data allow us to propose a mixed exploitation of sheep, with slaughtering aimed at both milk and meat production. On the one hand, the slaughter of animals in age classes C (6–12 m) and D (12–24 m) corresponds to the exploitation of meat before it reaches its optimal meat weight. In fact, the abundant presence of animals slaughtered before the first year of life would show an interest in the exploitation of tender meat. On the other hand, keeping animals beyond 24 months of age would correspond to the exploitation of milk and meat, since animals would be slaughtered when their productivity declines^59–62^. Payne's^63^ model for milk proposes the culling of newborn animals to avoid competition for milk between the offspring and the human group. However, these are theoretical models that would correspond to optimization for a single objective, and farmers oriented towards mixed meat and milk production tend to postpone slaughtering to exploit meat^64^. This would be the case in Tinj and Crno Vrilo, where milk would be exploited and, in addition, lambs would be kept after weaning for meat production. Dairy production has been previously proposed in the Adriatic from osteological analyses^10,31,56,57^ and was confirmed by ceramic residue analyses both in the area^65^ and throughout the Mediterranean^10,66^. Another aspect that is noteworthy is the abundant presence of animals slaughtered beyond 48 months at the Crno Vrilo site. This fact can have several explanations, the first of them would be related to the aforementioned exploitation of milk. In addition, animals can be kept alive longer to benefit from their manure production, as has been demonstrated for the Neolithic in other areas of the Mediterranean^67^. Finally, another explanation could be the search for herd security, minimizing herd fluctuations^41,42^.

In summary, the mortality profiles of Tinj and Crno Vrilo are very similar to each other. Considering that the differences are very slight, a similar exploitation strategy can be assumed for both sites. This strategy seems to be related to the conformation of the herds. Within this unity, the strategy could have multiple explanations such as those mentioned above (economic, social or cultural).

### Sheep reproduction

A main lambing period in early winter is different from what has been documented up to now in Neolithic Europe: in temperate Europe the dominant pattern is late winter/spring births^24,37^. Early winter births are documented in the Early and Middle Neolithic in the northwestern Mediterranean^21,22,68,69^ but in association with autumn lambing. Autumn lambing in the northwestern Mediterranean is explained by a capacity of Mediterranean breed for out-of-season breeding (an extended fertility period or less intense sexual rest)^70,71^ and manipulation by the herders who separate females and males and reintroduce males only in the spring to obtain autumn births.

Early winter as the main lambing period at Crno Vrilo and Tinj could be due to an extended period of fertility in these ewes (starting earlier compared to sheep at higher latitude in Europe) and no manipulation by the herders to delay breeding to the spring. An early mating (end of summer instead of autumn) could lead to early winter lambing. Winter births are common among current Mediterranean sheep husbandry systems and especially among Dalmatian autochthonous breeds^72–74^. The concentration of births in early winter could mean a rather good fertility rate (most females become pregnant at the beginning of the breeding period) while the (rather isolated) spring births could also be the sign of females failing in their pregnancy (losing their foetus for example) and getting fertilized again before the end of the fertility period.

The short lambing period is also a common livestock strategy among different pastoral groups, as mentioned by Tornero et al.^75^ for Southwest Asia, Bernus^76^ for the Sahel or as collected by sources for Mesopotamia at the end of the 3rd millennium BC^77^.

In summary, the data present in this study show that early winter lambing is favoured in Dalmatia, while autumn lambing is not documented until the introduction of sheep in the western Mediterranean. This could mean that there was already a capacity for a prolonged period of fertility in the ewes, but without manipulation by the herders. However, this would be advantageous for the herders. On the one hand, winter lambing may be partly related to the availability of pasture. The Mediterranean climate is characterized by dry summers and mild, wet winters. Thus, from autumn onwards, when rainfall increases^40^, pasture availability is higher in winter than in summer^78–80^. In addition, winter births favor milk production compared to spring births. It has been shown that climate can have adverse effects on milk production and milk quality^81,82^. The high temperatures of the Mediterranean climate negatively affect milk production, so the breeding season would be a fundamental aspect. This is the case for the autochthonous breeds of the study area, whose productivity increases in ewes lambing between autumn and winter^83^. The birth of most of the lambs in a short period of time would make it possible to organize the work within the annual cycle. The lambing period involves a lot of work for the herders, since they have to guarantee the survival of the lambs^84^. Moreover, this possible livestock calendar could be well articulated with the agricultural calendar, taking into account that the main crops of both sites are cereals^27,29^ that would be grown in winter and harvested in spring^85^. Finally, the anticipated mobility strategy proposed above would also be favoured by the birth of lambs in a short period of time. If the strategy of human groups is to plan mobility between areas, the concentration of births in a short space of time and in a period of difficult maritime mobility can be fundamental.

## Conclusion

The integrated archaeozoological, palaeoproteomic and isotopic results have allowed us to elucidate the practices of the first farmers in Dalmatia, linked to the Impressa Ware. The archaeozoological data indicated that livestock farming in the area was centred on caprine herds. This work, applying the ZooMS method for the first time in the region, has made it possible to reconstruct demographically the composition of these herds, showing that they were overwhelmingly composed of sheep, with very few goats present. Mortality profiles have shown a very similar exploitation of sheep for Tinj and Crno Vrilo, with meat and milk being the main products exploited. Finally, the data on sheep reproduction show that most of the births of sheep in both sites were concentrated at the beginning of winter, which contrasts with the data from the western Mediterranean for the Early Neolithic ^21,22,68,69^.

The integration of the results shows how in these two sites the early Dalmatian farmers, linked to the Impressa Ware, had a common animal economy. First of all, the conformation of the livestock herds at the two analysed sites was similar, with sheep as the main animal. Moreover, the sheep herds were exploited in a similar way, with a common breeding management. This common animal economy in both sites seems to indicate a cultural unity beyond the Impressa pottery. Early Dalmatian farmers had an economy based on the exploitation of sheep for products such as milk and meat with an early winter birth period which would have favoured production and would have been ideal for organising all the tasks of the annual cycle. Moreover, this common strategy could be related to the mobility practised by these early farming societies of the Impressa Ware throughout the Mediterranean. In any case, it will be important for future work to examine other sites around the Adriatic with the same methods, to assess whether our results are specific to these two Dalmatian sites or represent a consistent pattern of early Neolithic animal management across the wider region.

## Methods

### Mortality profiles

The estimation of the age at death was based on the study of dental remains, mainly from the analysis of tooth eruption and wear patterns. Data on tooth eruption and wear were recorded following Payne^63,86^, also using his age classes. In addition, these data were supplemented with metric data from the method of Helmer^87^ based on the decrease of the crown height (H/DT) with age.

Mortality profiles were then constructed following the method of Gerbault et al.^88^ which uses the Dirichlet distribution to construct histograms with Bayesian credible intervals that allow us to increase our ability to differentiate between age class representations within an archaeological assemblage. The 'rdirichlet' function of the R package LaplacesDemon (v. 4.1.3) was used for this purpose.

### ZooMS

ZooMS analysis was carried out on 193 mandibular and dental remains. Samples of between 10 and 30 mg were taken and demineralised by adding 250 µl of 0.6 M hydrochloric acid to the bone and left at 4 °C for approximately 2 days until the bone became flexible. To remove any possible contaminants, the remaining bone was rinsed once with 250 µl of 0.1 M sodium hydroxide and three times with 50 mM ammonium bicarbonate (NH4HCO3) buffer pH 8.0 (Ambic). The bone was then gelatinised in a heating block at 65 °C in 100 µl of Ambic for 1 h. A 50 µl aliquot of the supernatant was transferred to a new tube, to which 1 µl of 0.5 µg µl^−1^ trypsin was added, and the solution was left for 18 h in a 37 °C heating block. After stopping trypsin digestion by adding 1 µL of 5% trifluoroacetic acid (TFA), the peptides were extracted and purified using 100 µL of Pierce C18 ZipTips with washing (0.1% TFA and UHQ water) and conditioning (0.1% TFA in 50:50 acetonitrile and UHQ water) solutions. 1 µL of the sample was spotted in triplicate on a MALDI 384 plate with 1 μL of α-cyano-4-hydroxycinnamic acid matrix solution and air dried. MALDI analysis was carried out using a Bruker Ultraflex III MALDI-TOF mass spectrometer at the University of York. Replicates were averaged using the open-source software mMass (www.mmass.org;^81^) and compared to a database of known m/z markers^18–20^.

### Oxygen isotopes

For the study of reproduction, stable oxygen isotope analysis (δ^18^O) was performed on tooth enamel bioapatite. Following the procedure described in Balasse et al.^89^, sequential sampling was performed on the buccal side of the molar, in the middle lobe of M3. The enamel surface was cleaned by abrasion using a tungsten drill. Enamel was sampled sequentially perpendicularly to the tooth growth axis from the apex to the enamel-root junction using a diamond drill bit. Samples were spaced at 1–1.5 mm intervals. A low magnification lens (× 3) was used throughout the sampling process. The samples were located in the tooth crown using their distance from the enamel-root junction. The samples were then pre-treated for 4 h in 0.1 M acetic acid [CH3COOH] (0.1 ml of solution/0.1 mg of sample). The weight loss caused by this pretreatment was 29.5 ± 6%.

The pre-treated enamel powders were analysed on a Kiel IV device connected to a DeltaVAdvantage IRMS. The accuracy and precision of the measurements were verified using an internal laboratory calcium carbonate standard (Marbre LM standardised according to the international standard NBS 19). The results are expressed in V-PDB. The analytical precision, estimated from four to eight Marbre LM analyses, was on average 0.03‰ for δ^18^O values and 0.02‰ for δ^13^C values. Over the period of analysis of the enamel samples, the analysis of 102 Marbre LM gave an average δ^18^O value of − 2.03 ± 0.1‰ (expected value = − 1.83‰).

The δ^18^O sequences were modelled using an equation derived from a cosine function described in Balasse et al.^90^ using four parameters: the position of the maximum value of δ^18^O (× 0); the cycle period (X; distance over which an annual cycle was recorded); the signal amplitude (A) and the mean (M). The cycle period (X) was used to normalise × 0 to remove inter-individual variability in tooth size^83^. The ratio × 0/X varies with season of birth. Season of birth is estimated by comparison with reference × 0/X ratios obtained in modern sheep^24,37^. All results are shown using a circular representation to reflect the cyclical nature of seasonality^24^.

## Supplementary Information

Supplementary Information 1.

Supplementary Information 2.

Supplementary Information 3.

## Data Availability

All data generated or analyzed during this study are included in this published article [and its supplementary information files].
