# Supplementary material for: Early Dalmatian farmers specialized in sheep husbandry
Source: Sci Rep. 2023 Jun 26;13:10355. doi: 10.1038/s41598-023-37516-z (PMC10293258; doi:10.1038/s41598-023-37516-z)
Supplement: Supplementary file 1 — Supplementary Information 1. [file 41598_2023_37516_MOESM1_ESM.docx]

**Suplementary materials 1: Chronological data of Tinj and Crno Vrlo**

In these supplementary materials, dates from the Tinj and Crno Vrlo sites are presented. As the Tinj dates published so far were not AMS and had a large deviation, a new bone from the site has been dated to confirm the chronology.


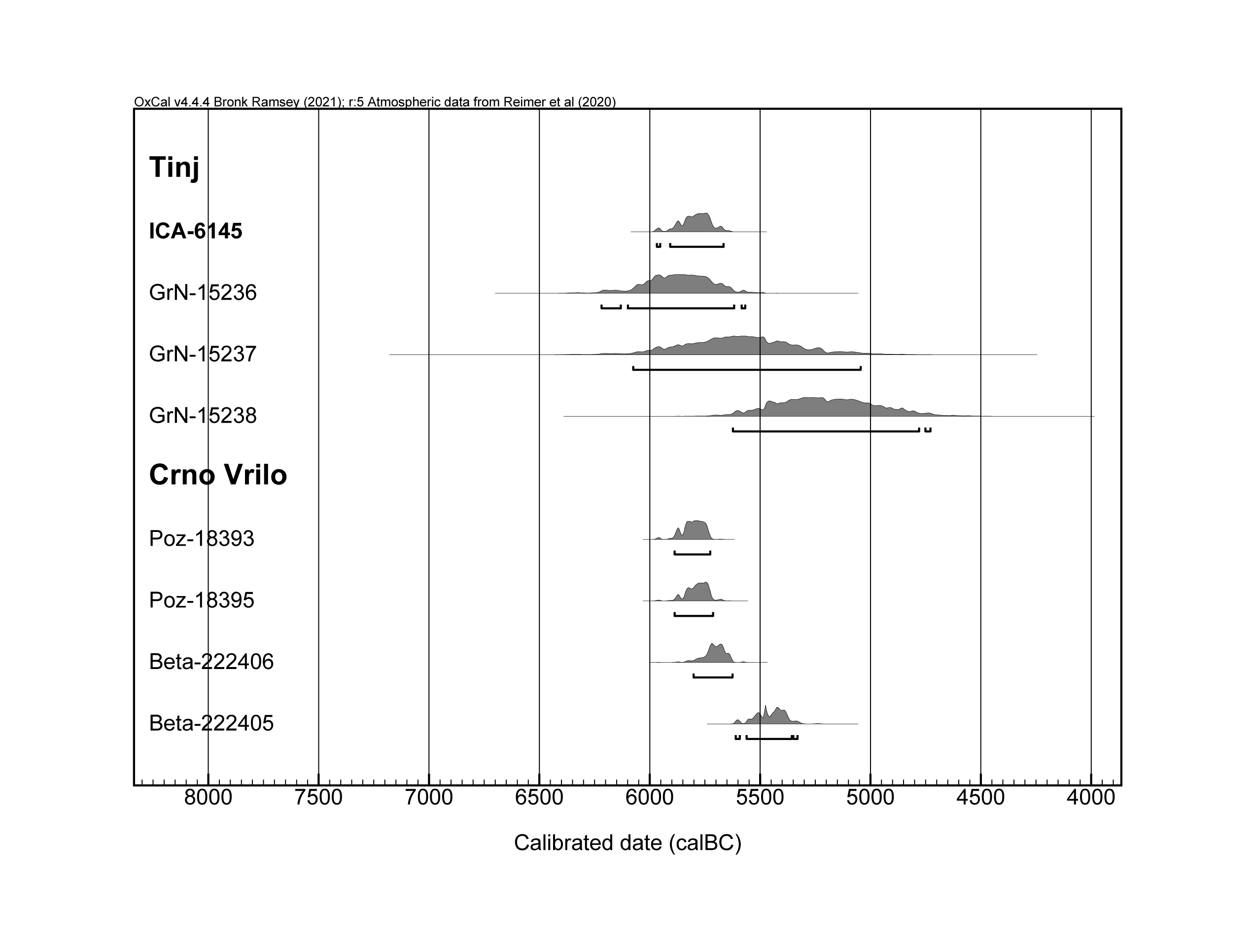


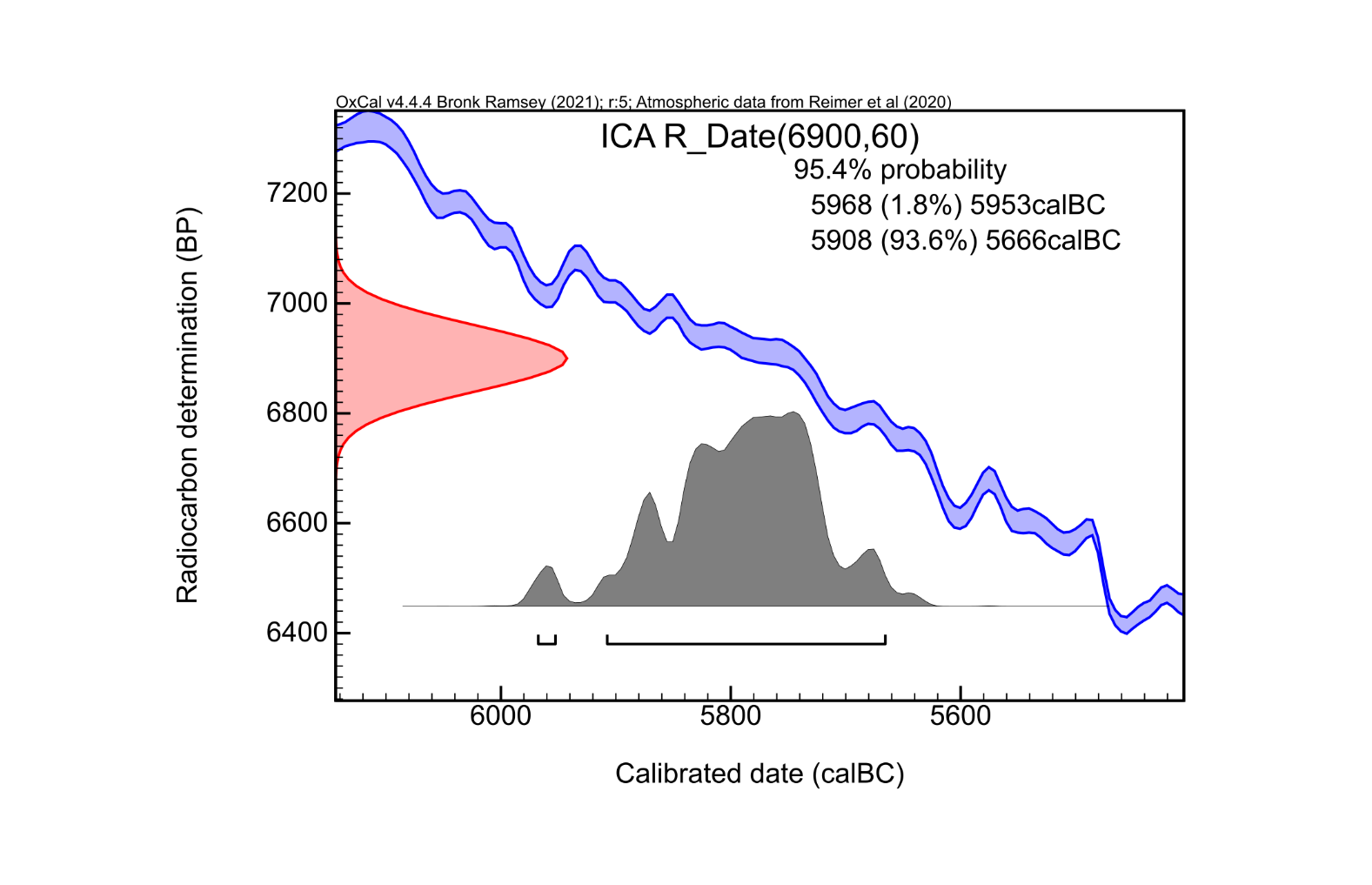


New dating for this paper in Tinj
