# Supplementary material for: Early Dalmatian farmers specialized in sheep husbandry
Source: Sci Rep. 2023 Jun 26;13:10355. doi: 10.1038/s41598-023-37516-z (PMC10293258; doi:10.1038/s41598-023-37516-z)
Supplement: Supplementary file 3 — Supplementary Information 3. [file 41598_2023_37516_MOESM3_ESM.docx]

**Supplementary materials 3: oxygen isotopes results**

| **sample** | **mm from ERJ** | **δ^18^O_VPDB_ (‰)** |
| --- | --- | --- |
| T76 M3 1 | 35 | -1.043 |
| T76 M3 2 | 33.45 | -1.191 |
| T76 M3 3 | 31.91 | -0.949 |
| T76 M3 4 | 30.3 | -0.689 |
| T76 M3 5 | 28.82 | -0.764 |
| T76 M3 6 | 27.54 | -0.519 |
| T76 M3 7 | 26.08 | -0.634 |
| T76 M3 8 | 24.56 | -0.727 |
| T76 M3 9 | 22.71 | -1.173 |
| T76 M3 10 | 21.67 | -1.303 |
| T76 M3 11 | 20.17 | -1.749 |
| T76 M3 12 | 18.4 | -2.587 |
| T76 M3 13 | 16.75 | -3.285 |
| T76 M3 14 | 14.93 | -4.002 |
| T76 M3 15 | 13.55 | -4.689 |
| T76 M3 16 | 11.65 | -3.978 |
| T76 M3 17 | 10.31 | -3.383 |
| T76 M3 18 | 8.59 | -1.898 |
| T76 M3 19 | 7.13 | -1.007 |
| T76 M3 20 | 5.72 | -1.637 |
|  |  |  |
| **sample** | **mm from ERJ** | **δ^18^O_VPDB_ (‰)** |
| T8 M3 1 | 21.14 | -1.305 |
| T8 M3 2 | 19.53 | -0.820 |
| T8 M3 3 | 18.3 | -1.225 |
| T8 M3 4 | 17.09 | -0.798 |
| T8 M3 5 | 15.19 | -0.961 |
| T8 M3 6 | 14.05 | -0.654 |
| T8 M3 7 | 12.66 | -0.406 |
| T8 M3 8 | 10.76 | -1.286 |
| T8 M3 9 | 9.36 | -1.673 |
| T8 M3 10 | 7.56 | -2.587 |
| T8 M3 11 | 5.78 | -2.775 |
| T8 M3 12 | 4.59 | -1.531 |
| T8 M3 13 | 2.39 | -1.160 |
|  |  |  |
| **sample** | **mm from ERJ** | **δ^18^O_VPDB_ (‰)** |
| T48 M3 1 | 25.61 | -2.049 |
| T48 M3 2 | 24.3 | -2.812 |
| T48 M3 3 | 22.79 | -3.327 |
| T48 M3 4 | 21.07 | -3.932 |
| T48 M3 5 | 19.47 | -3.751 |
| T48 M3 6 | 17.64 | -2.484 |
| T48 M3 7 | 15.76 | -2.201 |
| T48 M3 8 | 14.08 | -1.630 |
| T48 M3 9 | 12.42 | -1.897 |
| T48 M3 10 | 10.78 | -2.086 |
| T48 M3 11 | 9.35 | -1.898 |
| T48 M3 12 | 7.6 | -1.604 |
| T48 M3 13 | 6.56 | -2.409 |
| T48 M3 14 | 5.01 | -2.393 |
| T48 M3 15 | 2.8 | -3.485 |
|  |  |  |
| **sample** | **mm from ERJ** | **δ^18^O_VPDB_ (‰)** |
| T47 M3 1 | 25.98 | -1.753 |
| T47 M3 2 | 24.43 | -1.579 |
| T47 M3 3 | 23.39 | -0.274 |
| T47 M3 4 | 21.57 | 0.538 |
| T47 M3 5 | 20.32 | 0.352 |
| T47 M3 6 | 18.4 | -0.295 |
| T47 M3 7 | 16.83 | -1.012 |
| T47 M3 8 | 15.21 | -0.809 |
| T47 M3 9 | 13.47 | -1.737 |
| T47 M3 10 | 12.39 | -2.042 |
| T47 M3 11 | 10.39 | -2.552 |
| T47 M3 12 | 9.17 | -2.094 |
| T47 M3 13 | 8.04 | -1.523 |
| T47 M3 14 | 7.31 | -0.572 |
| T47 M3 15 | 5.61 | -0.726 |
| T47 M3 16 | 4.82 | -1.006 |
| T47 M3 17 | 3.1 | -0.868 |
|  |  |  |
| **sample** | **mm from ERJ** | **δ^18^O_VPDB_ (‰)** |
| CV69 M3 1 | 30.69 | -3.820 |
| CV69 M3 2 | 28.82 | -4.293 |
| CV69 M3 3 | 27.09 | -4.654 |
| CV69 M3 4 | 25.48 | -4.362 |
| CV69 M3 5 | 24 | -4.219 |
| CV69 M3 6 | 22.42 | -3.499 |
| CV69 M3 7 | 21 | -2.969 |
| CV69 M3 8 | 19.52 | -1.930 |
| CV69 M3 9 | 17.88 | -1.533 |
| CV69 M3 10 | 16.14 | -1.205 |
| CV69 M3 11 | 14.35 | -0.266 |
| CV69 M3 12 | 12.65 | 0.313 |
| CV69 M3 13 | 11.34 | 0.845 |
| CV69 M3 14 | 9.54 | 0.861 |
| CV69 M3 15 | 7.87 | -0.440 |
| CV69 M3 16 | 6.55 | -2.375 |
| CV69 M3 17 | 4.05 | -4.059 |
|  |  |  |
| **sample** | **mm from ERJ** | **δ^18^O_VPDB_ (‰)** |
| CV52 M3 1 | 28.35 | -1.874 |
| CV52 M3 2 | 27.15 | -1.693 |
| CV52 M3 3 | 25.55 | -2.119 |
| CV52 M3 4 | 23.99 | -2.017 |
| CV52 M3 5 | 22.56 | -1.969 |
| CV52 M3 6 | 20.78 | -2.071 |
| CV52 M3 7 | 19.37 | -3.396 |
| CV52 M3 8 | 17.76 | -3.756 |
| CV52 M3 9 | 16.38 | -4.279 |
| CV52 M3 10 | 14.42 | -4.471 |
| CV52 M3 11 | 13.03 | -4.649 |
| CV52 M3 12 | 11.72 | -4.869 |
| CV52 M3 13 | 10.14 | -3.941 |
| CV52 M3 14 | 8.33 | -3.069 |
| CV52 M3 15 | 7.15 | -1.797 |
| CV52 M3 16 | 6.04 | -2.084 |
| CV52 M3 17 | 5.03 | -1.577 |
| CV52 M3 18 | 3.2 | -1.840 |
|  |  |  |
| **sample** | **mm from ERJ** | **δ^18^O_VPDB_ (‰)** |
| CV136 M3 1 | 35.98 | -1.393 |
| CV136 M3 2 | 34.19 | -0.795 |
| CV136 M3 3 | 32.41 | -0.746 |
| CV136 M3 4 | 30.61 | -0.858 |
| CV136 M3 5 | 28.55 | -0.292 |
| CV136 M3 6 | 26.86 | -0.871 |
| CV136 M3 7 | 25.22 | 0.112 |
| CV136 M3 8 | 23.94 | -0.395 |
| CV136 M3 9 | 22.59 | -0.386 |
| CV136 M3 10 | 21.01 | -1.769 |
| CV136 M3 11 | 19.27 | -2.689 |
| CV136 M3 12 | 17.68 | -3.583 |
| CV136 M3 13 | 16.11 | -4.613 |
| CV136 M3 14 | 14.85 | -5.351 |
| CV136 M3 15 | 13.65 | -4.838 |
| CV136 M3 16 | 12.27 | -4.412 |
| CV136 M3 17 | 10.85 | -3.432 |
| CV136 M3 18 | 9.43 | -2.206 |
| CV136 M3 19 | 7.78 | -0.722 |
| CV136 M3 20 | 6.1 | -0.653 |
| CV136 M3 21 | 4.42 | -0.439 |
|  |  |  |
| **sample** | **mm from ERJ** | **δ^18^O_VPDB_ (‰)** |
| CV72 M3 1 | 30.8 | -0.291 |
| CV72 M3 2 | 29.5 | -0.078 |
| CV72 M3 3 | 27.34 | -0.513 |
| CV72 M3 4 | 25.95 | -0.153 |
| CV72 M3 5 | 24.61 | -0.125 |
| CV72 M3 6 | 23.08 | -0.546 |
| CV72 M3 7 | 21.5 | -1.515 |
| CV72 M3 8 | 19.9 | -1.889 |
| CV72 M3 9 | 18.32 | -2.804 |
| CV72 M3 10 | 16.78 | -3.154 |
| CV72 M3 11 | 15.41 | -3.190 |
| CV72 M3 12 | 13.79 | -2.916 |
| CV72 M3 13 | 12.14 | -2.230 |
| CV72 M3 14 | 10.89 | -1.561 |
| CV72 M3 15 | 9.36 | -1.249 |
| CV72 M3 16 | 7.56 | -0.753 |
| CV72 M3 17 | 5.77 | -0.902 |
|  |  |  |
| **sample** | **mm from ERJ** | **δ^18^O_VPDB_ (‰)** |
| CV125 M3 1 | 32.95 | -0.060 |
| CV125 M3 2 | 31.48 | -0.337 |
| CV125 M3 3 | 29.71 | 0.171 |
| CV125 M3 4 | 28.17 | 0.248 |
| CV125 M3 5 | 26.96 | -0.043 |
| CV125 M3 6 | 25.55 | -0.507 |
| CV125 M3 7 | 23.73 | -0.993 |
| CV125 M3 8 | 21.78 | -1.742 |
| CV125 M3 9 | 20.79 | -2.661 |
| CV125 M3 10 | 19.01 | -3.802 |
| CV125 M3 11 | 17.81 | -4.687 |
| CV125 M3 12 | 16.28 | -5.113 |
| CV125 M3 13 | 14.55 | -5.119 |
| CV125 M3 14 | 12.89 | -4.478 |
| CV125 M3 15 | 11.21 | -3.644 |
| CV125 M3 16 | 9.46 | -2.996 |
| CV125 M3 17 | 8.11 | -2.402 |
| CV125 M3 18 | 6.52 | -2.005 |
| CV125 M3 19 | 5.46 | -1.423 |
|  |  |  |
| **sample** | **mm from ERJ** | **δ^18^O_VPDB_ (‰)** |
| CV32 M3 1 | 27.21 | -4.070 |
| CV32 M3 2 | 25.56 | -4.043 |
| CV32 M3 3 | 23.72 | -3.798 |
| CV32 M3 4 | 21.92 | -3.537 |
| CV32 M3 5 | 20.22 | -3.389 |
| CV32 M3 6 | 18.78 | -2.815 |
| CV32 M3 7 | 16.78 | -2.563 |
| CV32 M3 8 | 15.19 | -1.924 |
| CV32 M3 9 | 13.6 | -1.919 |
| CV32 M3 10 | 12.2 | -2.682 |
| CV32 M3 11 | 10.78 | -2.825 |
| CV32 M3 12 | 8.95 | -4.042 |
| CV32 M3 13 | 7.55 | -4.425 |
| CV32 M3 14 | 6.04 | -4.093 |
| CV32 M3 15 | 4.4 | -3.282 |
| CV32 M3 16 | 2.76 | -2.384 |
|  |  |  |
| **sample** | **mm from ERJ** | **δ^18^O_VPDB_ (‰)** |
| CV122 M3 1 | 34.78 | -4.128 |
| CV122 M3 2 | 33 | -4.974 |
| CV122 M3 3 | 31.47 | -4.600 |
| CV122 M3 4 | 29.7 | -4.855 |
| CV122 M3 5 | 28.05 | -4.025 |
| CV122 M3 6 | 26.86 | -3.667 |
| CV122 M3 7 | 25.36 | -2.842 |
| CV122 M3 8 | 23.92 | -2.487 |
| CV122 M3 9 | 22.43 | -1.777 |
| CV122 M3 10 | 21.01 | -1.482 |
| CV122 M3 11 | 19.59 | -1.598 |
| CV122 M3 12 | 18.17 | -1.145 |
| CV122 M3 13 | 16.59 | -1.488 |
| CV122 M3 14 | 14.24 | -1.121 |
| CV122 M3 15 | 12.71 | -0.777 |
| CV122 M3 16 | 11.34 | -1.867 |
| CV122 M3 17 | 9.27 | -1.634 |
| CV122 M3 18 | 8 | -2.944 |
| CV122 M3 19 | 5.97 | -3.713 |
| CV122 M3 20 | 3.84 | -3.820 |
|  |  |  |
| **sample** | **mm from ERJ** | **δ^18^O_VPDB_ (‰)** |
| CV137 M3 1 | 36.42 | -1.481 |
| CV137 M3 2 | 34.62 | -1.804 |
| CV137 M3 3 | 33.06 | -0.831 |
| CV137 M3 4 | 31.48 | -0.553 |
| CV137 M3 5 | 29.91 | -0.318 |
| CV137 M3 6 | 27.96 | -0.344 |
| CV137 M3 7 | 26.66 | -0.535 |
| CV137 M3 8 | 25.03 | -0.639 |
| CV137 M3 9 | 23.16 | -1.294 |
| CV137 M3 10 | 21.19 | -2.034 |
| CV137 M3 11 | 19.15 | -2.988 |
| CV137 M3 12 | 17.79 | -3.632 |
| CV137 M3 13 | 15.86 | -3.796 |
| CV137 M3 14 | 14.01 | -4.047 |
| CV137 M3 15 | 12.07 | -3.731 |
| CV137 M3 16 | 10.27 | -3.505 |
| CV137 M3 17 | 8.68 | -2.342 |
| CV137 M3 18 | 6.97 | -0.891 |
| CV137 M3 19 | 5.42 | 0.039 |
|  |  |  |
| **sample** | **mm from ERJ** | **δ^18^O_VPDB_ (‰)** |
| CV113 M3 1 | 31.83 | 3.046 |
| CV113 M3 2 | 30.49 | 3.108 |
| CV113 M3 3 | 29.16 | 2.204 |
| CV113 M3 4 | 27.59 | 1.979 |
| CV113 M3 5 | 25.68 | 1.045 |
| CV113 M3 6 | 23.6 | -0.304 |
| CV113 M3 7 | 21.91 | -1.423 |
| CV113 M3 8 | 20.14 | -2.465 |
| CV113 M3 9 | 18.75 | -3.308 |
| CV113 M3 10 | 16.81 | -3.505 |
| CV113 M3 11 | 15.34 | -3.701 |
| CV113 M3 12 | 13.75 | -2.913 |
| CV113 M3 13 | 11.9 | -2.532 |
| CV113 M3 14 | 10.24 | -1.813 |
| CV113 M3 15 | 8.3 | -0.992 |
| CV113 M3 16 | 6.78 | -0.824 |
| CV113 M3 17 | 5.17 | 0.148 |
|  |  |  |
| **sample** | **mm from ERJ** | **δ^18^O_VPDB_ (‰)** |
| CV97 M3 1 | 32.71 | -0.110 |
| CV97 M3 2 | 31.13 | 0.040 |
| CV97 M3 3 | 29.45 | 0.140 |
| CV97 M3 4 | 28.03 | 0.157 |
| CV97 M3 5 | 26.25 | -0.165 |
| CV97 M3 6 | 25.11 | -0.640 |
| CV97 M3 7 | 23.12 | -0.822 |
| CV97 M3 8 | 21.57 | -1.528 |
| CV97 M3 9 | 19.63 | -2.784 |
| CV97 M3 10 | 18.06 | -2.863 |
| CV97 M3 11 | 16.5 | -3.361 |
| CV97 M3 12 | 14.54 | -3.662 |
| CV97 M3 13 | 12.99 | -3.226 |
| CV97 M3 14 | 10.83 | -3.078 |
| CV97 M3 15 | 9.67 | -2.700 |
| CV97 M3 16 | 7.96 | -2.058 |
| CV97 M3 17 | 6.45 | -1.324 |
| CV97 M3 18 | 4.79 | -0.121 |
|  |  |  |
| **sample** | **mm from ERJ** | **δ^18^O_VPDB_ (‰)** |
| CV41 M3 1 | 30.01 | -0.641 |
| CV41 M3 2 | 27.76 | -0.351 |
| CV41 M3 3 | 26.28 | -0.783 |
| CV41 M3 4 | 24.42 | -1.173 |
| CV41 M3 5 | 22.72 | -1.596 |
| CV41 M3 6 | 21.03 | -1.821 |
| CV41 M3 7 | 19.48 | -2.518 |
| CV41 M3 8 | 17.66 | -3.237 |
| CV41 M3 9 | 16.57 | -3.761 |
| CV41 M3 10 | 14.69 | -4.222 |
| CV41 M3 11 | 13.14 | -4.659 |
| CV41 M3 12 | 11.89 | -4.076 |
| CV41 M3 13 | 9.53 | -4.136 |
| CV41 M3 14 | 8.14 | -2.878 |
| CV41 M3 15 | 6.48 | -2.237 |
| CV41 M3 16 | 5.35 | -1.557 |
| CV41 M3 17 | 3.8 | -1.142 |
|  |  |  |
| **sample** | **mm from ERJ** | **δ^18^O_VPDB_ (‰)** |
| T44 M3 1 | 23.55 | -3.428 |
| T44 M3 2 | 21.27 | -4.173 |
| T44 M3 3 | 20.58 | -3.899 |
| T44 M3 4 | 19.32 | -3.832 |
| T44 M3 5 | 17.65 | -3.446 |
| T44 M3 6 | 15.96 | -3.167 |
| T44 M3 7 | 14.65 | -2.824 |
| T44 M3 8 | 12.96 | -2.468 |
| T44 M3 9 | 11.36 | -1.844 |
| T44 M3 10 | 9.97 | -1.637 |
| T44 M3 11 | 8.74 | -0.713 |
| T44 M3 12 | 7.05 | -0.646 |
| T44 M3 13 | 5.58 | -0.573 |
| T44 M3 14 | 4.18 | -1.201 |
| T44 M3 15 | 2.99 | -1.629 |
|  |  |  |
| **sample** | **mm from ERJ** | **δ^18^O_VPDB_ (‰)** |
| CV129 M3 1 | 34.42 | -1.820 |
| CV129 M3 2 | 32.6 | -0.922 |
| CV129 M3 3 | 30.92 | -0.310 |
| CV129 M3 4 | 29.2 | -0.085 |
| CV129 M3 5 | 27.61 | 0.382 |
| CV129 M3 6 | 25.88 | 0.718 |
| CV129 M3 7 | 24.33 | 0.644 |
| CV129 M3 8 | 22.92 | 0.286 |
| CV129 M3 9 | 21.28 | -0.156 |
| CV129 M3 10 | 20.16 | 0.337 |
| CV129 M3 11 | 18.17 | -1.819 |
| CV129 M3 12 | 16.39 | -3.135 |
| CV129 M3 13 | 14.82 | -3.901 |
| CV129 M3 14 | 13.36 | -3.254 |
| CV129 M3 15 | 11.15 | -3.508 |
| CV129 M3 16 | 9.63 | -2.574 |
| CV129 M3 17 | 7.65 | -1.750 |
| CV129 M3 18 | 6.14 | -1.456 |
|  |  |  |
| **sample** | **mm from ERJ** | **δ^18^O_VPDB_ (‰)** |
| CV112 M3 1 | 29.53 | -0.572 |
| CV112 M3 2 | 28.09 | -1.548 |
| CV112 M3 3 | 25.99 | -1.964 |
| CV112 M3 4 | 24.19 | -2.303 |
| CV112 M3 5 | 22.16 | -3.758 |
| CV112 M3 6 | 20.65 | -4.409 |
| CV112 M3 7 | 19.04 | -3.900 |
| CV112 M3 8 | 17.45 | -3.010 |
| CV112 M3 9 | 15.96 | -1.789 |
| CV112 M3 10 | 13.68 | -1.157 |
| CV112 M3 11 | 12.45 | -0.650 |
| CV112 M3 12 | 10.56 | -0.667 |
| CV112 M3 13 | 8.95 | -0.341 |
| CV112 M3 14 | 7.56 | -0.639 |
| CV112 M3 15 | 5.86 | -1.035 |
| CV112 M3 16 | 3.7 | -2.711 |
|  |  |  |
| **sample** | **mm from ERJ** | **δ^18^O_VPDB_ (‰)** |
| T63 M3 1 | 34.84 | 0.139 |
| T63 M3 2 | 32.76 | 0.546 |
| T63 M3 3 | 31.26 | 0.683 |
| T63 M3 4 | 29.45 | 0.256 |
| T63 M3 5 | 28.01 | -0.643 |
| T63 M3 6 | 26.74 | -0.660 |
| T63 M3 7 | 25.06 | -1.358 |
| T63 M3 8 | 23.49 | -2.356 |
| T63 M3 9 | 21.66 | -3.070 |
| T63 M3 10 | 19.7 | -3.476 |
| T63 M3 11 | 18.1 | -3.733 |
| T63 M3 12 | 16.73 | -3.917 |
| T63 M3 13 | 14.65 | -3.244 |
| T63 M3 14 | 13.08 | -2.596 |
| T63 M3 15 | 11.3 | -1.389 |
| T63 M3 16 | 8.9 | -0.893 |
| T63 M3 17 | 7.82 | -1.403 |
| T63 M3 18 | 6.15 | -0.571 |
| T63 M3 19 | 4.48 | -0.581 |
|  |  |  |
| **sample** | **mm from ERJ** | **δ^18^O_VPDB_ (‰)** |
| CV42 M3 1 | 31.54 | -0.714 |
| CV42 M3 2 | 30.37 | -1.063 |
| CV42 M3 3 | 29.09 | -2.030 |
| CV42 M3 4 | 27.21 | -2.339 |
| CV42 M3 5 | 25.28 | -3.180 |
| CV42 M3 6 | 23.66 | -3.777 |
| CV42 M3 7 | 21.77 | -3.776 |
| CV42 M3 8 | 20.18 | -3.808 |
| CV42 M3 9 | 18.77 | -3.458 |
| CV42 M3 10 | 16.76 | -2.763 |
| CV42 M3 11 | 15.2 | -2.364 |
| CV42 M3 12 | 13.24 | -1.067 |
| CV42 M3 13 | 11.51 | -0.799 |
| CV42 M3 14 | 9.68 | -0.313 |
| CV42 M3 15 | 8 | -0.451 |
| CV42 M3 16 | 6.6 | -0.526 |
| CV42 M3 17 | 5.14 | -1.231 |
|  |  |  |
| **sample** | **mm from ERJ** | **δ^18^O_VPDB_ (‰)** |
| T9 M3 1 | 36.49 | 0.121 |
| T9 M3 2 | 34.61 | 0.184 |
| T9 M3 3 | 32.68 | -0.320 |
| T9 M3 4 | 30.85 | -0.376 |
| T9 M3 5 | 28.83 | -0.750 |
| T9 M3 6 | 27.02 | -1.298 |
| T9 M3 7 | 24.95 | -2.361 |
| T9 M3 8 | 23.21 | -3.009 |
| T9 M3 9 | 21.52 | -3.715 |
| T9 M3 10 | 19.87 | -3.967 |
| T9 M3 11 | 18.3 | -4.355 |
| T9 M3 12 | 16.63 | -4.188 |
| T9 M3 13 | 15.01 | -3.587 |
| T9 M3 14 | 13.08 | -2.215 |
| T9 M3 15 | 11.78 | -1.977 |
| T9 M3 16 | 10.32 | -0.933 |
| T9 M3 17 | 8.72 | -0.670 |
| T9 M3 18 | 6.68 | -1.817 |
|  |  |  |
| **sample** | **mm from ERJ** | **δ^18^O_VPDB_ (‰)** |
| CV43 M3 1 | 32.41 | 0.680 |
| CV43 M3 2 | 30.62 | 1.095 |
| CV43 M3 3 | 28.9 | 1.094 |
| CV43 M3 4 | 26.95 | 0.837 |
| CV43 M3 5 | 24.92 | -0.149 |
| CV43 M3 6 | 23.5 | -1.528 |
| CV43 M3 7 | 21.57 | -2.643 |
| CV43 M3 8 | 19.8 | -3.599 |
| CV43 M3 9 | 17.91 | -4.173 |
| CV43 M3 10 | 15.86 | -3.672 |
| CV43 M3 11 | 13.97 | -3.394 |
| CV43 M3 12 | 12.03 | -3.156 |
| CV43 M3 13 | 10.23 | -2.136 |
| CV43 M3 14 | 8.6 | -1.356 |
| CV43 M3 15 | 6.68 | -0.365 |
| CV43 M3 16 | 5.13 | -0.363 |
|  |  |  |
| **sample** | **mm from ERJ** | **δ^18^O_VPDB_ (‰)** |
| CV149 M3 1 | 36.54 | -1.830 |
| CV149 M3 2 | 34.87 | -1.499 |
| CV149 M3 3 | 33.25 | -1.681 |
| CV149 M3 4 | 31.59 | -0.774 |
| CV149 M3 5 | 29.85 | -0.569 |
| CV149 M3 6 | 27.99 | -0.398 |
| CV149 M3 7 | 26.42 | -0.649 |
| CV149 M3 8 | 25.05 | -0.683 |
| CV149 M3 9 | 23.54 | -0.267 |
| CV149 M3 10 | 21.7 | -0.602 |
| CV149 M3 11 | 20.11 | -0.962 |
| CV149 M3 12 | 18.58 | -1.325 |
| CV149 M3 13 | 16.77 | -1.920 |
| CV149 M3 14 | 14.82 | -2.479 |
| CV149 M3 15 | 13.19 | -2.863 |
| CV149 M3 16 | 11.17 | -2.246 |
| CV149 M3 17 | 9.7 | -1.300 |
| CV149 M3 18 | 7.78 | -0.290 |
| CV149 M3 19 | 6.1 | -0.029 |


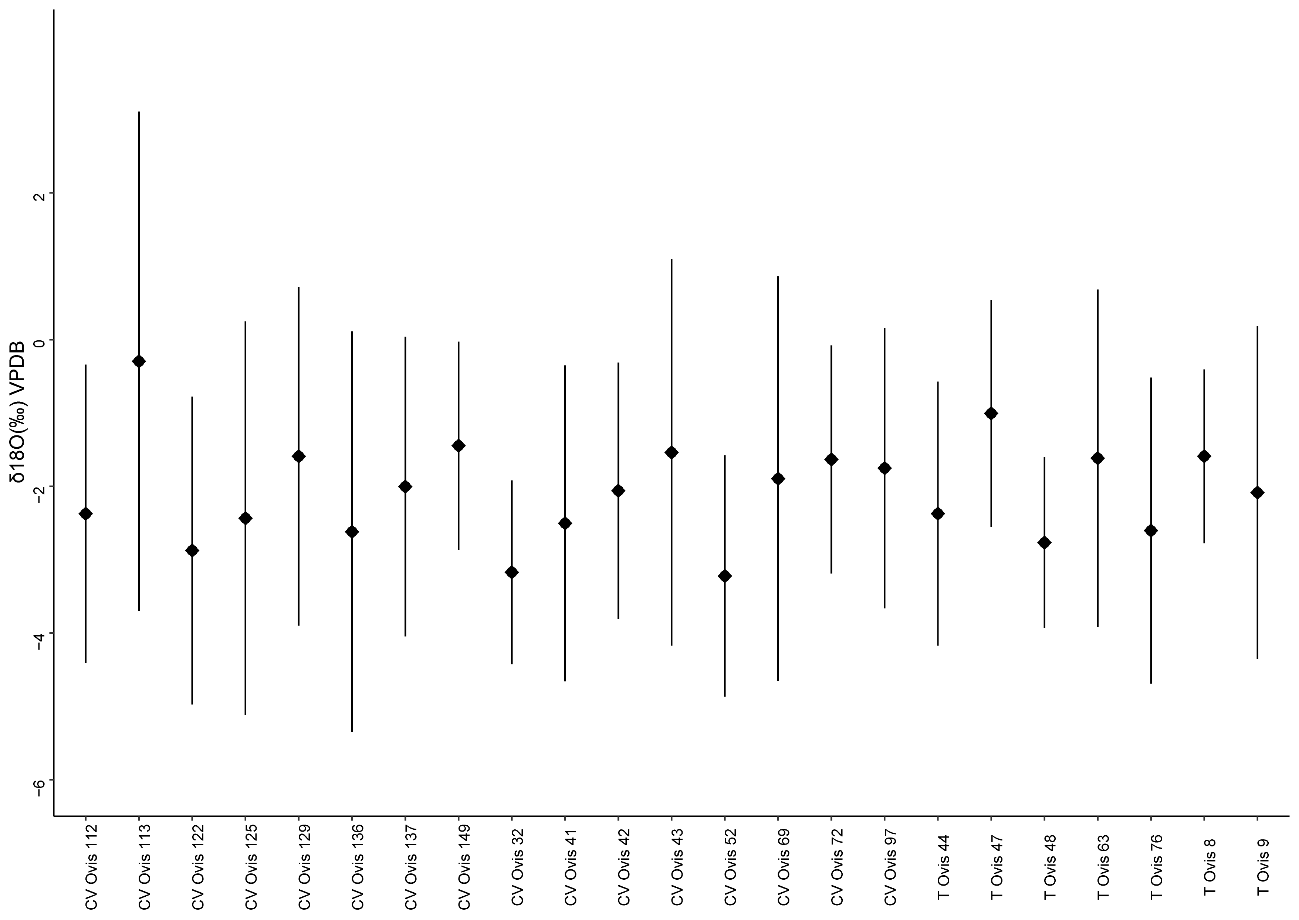


| **Nom** | **T76 M3** | **T44 M3** | **T9 M3** | **T63 M3** |
| --- | --- | --- | --- | --- |
| X | 23.876 | 27.117 | 29.940 | 30.170 |
| A | 1.902 | 1.592 | 2.009 | 1.980 |
| x0 | 2.280 | 6.686 | 3.777 | 32.491 |
| M | -2.115 | -2.365 | -2.149 | -1.623 |
|  |  |  |  |  |
| x0/X | 0.095 | 0.247 | 0.126 | 0.077 |

| **Nom** | **CV69 M3** | **CV52 M3** | **CV136 M3** | **CV72 M3** | **CV125 M3** |
| --- | --- | --- | --- | --- | --- |
| X | 27.054 | 21.802 | 22.661 | 23.828 | 27.585 |
| A | 2.603 | 1.551 | 2.427 | 1.470 | 2.545 |
| x0 | 12.637 | 3.217 | 3.406 | 3.671 | 0.892 |
| M | -2.134 | -3.121 | -2.269 | -1.492 | -2.347 |
|  |  |  |  |  |  |
| x0/X | 0.467 | 0.148 | 0.150 | 0.154 | 0.032 |

| **Nom** | **CV122 M3** | **CV137M3** | **CV113 M3** | **CV97 M3** | **CV41 M3** |
| --- | --- | --- | --- | --- | --- |
| X | 33.369 | 25.746 | 32.680 | 28.967 | 27.527 |
| A | 1.873 | 2.021 | 3.296 | 1.916 | 1.935 |
| x0 | 16.028 | 2.145 | 32.055 | 29.082 | 27.228 |
| M | -2.792 | -2.068 | -0.242 | -1.630 | -2.371 |
|  |  |  |  |  |  |
| x0/X | 0.480 | 0.083 | 0.981 | 1.004 | 0.989 |

| **Nom** | **CV129 M3** | **CV112 M3** | **CV42 M3** | **CV43 M3** |
| --- | --- | --- | --- | --- |
| X | 26.572 | 21.513 | 25.431 | 28.761 |
| A | 2.152 | 1.788 | 1.769 | 2.544 |
| x0 | 25.984 | 9.980 | 8.936 | 30.360 |
| M | -1.449 | -2.048 | -2.139 | -1.680 |
|  |  |  |  |  |
| x0/X | 0.978 | 0.464 | 0.351 | 0.056 |
